# Donor selection for KIR alloreactivity is associated with superior survival in haploidentical transplant with PTCy

**DOI:** 10.3389/fimmu.2022.1033871

**Published:** 2022-10-13

**Authors:** Jun Zou, Piyanuch Kongtim, Samer A. Srour, Uri Greenbaum, Johannes Schetelig, Falk Heidenreich, Henning Baldauf, Brandt Moore, Supawee Saengboon, Yudith Carmazzi, Gabriela Rondon, Qing Ma, Katayoun Rezvani, Elizabeth J. Shpall, Richard E. Champlin, Stefan O. Ciurea, Kai Cao

**Affiliations:** ^1^ Department of Laboratory Medicine, The University of Texas MD Anderson Cancer Center, Houston, TX, United States; ^2^ Division of Hematology/Oncology, Department of Medicine, Chao Family Comprehensive Cancer Center, University of California, Irvine, Orange, CA, United States; ^3^ Center of Excellence in Applied Epidemiology, Faculty of Medicine, Thammasat University, Pathumthani, Thailand; ^4^ Department of Stem Cell Transplantation and Cellular Therapy, The University of Texas MD Anderson Cancer Center, Houston, TX, United States; ^5^ Department of Hematology, Soroka University Medical Center, and Faculty of Health Sciences, Ben Gurion University of the Negev, Beer Sheva, Israel; ^6^ Department of Internal Medicine I, University Hospital Carl Gustav Carus, TU Dresden, Dresden, Germany; ^7^ DKMS gemeinnützige GmbH, Tübingen, Germany; ^8^ Department of Hematopoietic Biology and Malignancy, The University of Texas MD Anderson Cancer Center, Houston, TX, United States

**Keywords:** haplo-HSCT, NK cell alloreactivity, KIR, donor selection, overall survival, progression-free survival

## Abstract

With the continuous increase in the use of haploidentical donors for transplantation, the selection of donors becomes increasingly important. Haploidentical donors have been selected primarily based on clinical characteristics, while the effects of killer cell immunoglobulin-like receptors (KIRs) on outcomes of haploidentical-hematopoietic stem cell transplantation (haplo-HSCT) with post-transplant cyclophosphamide (PTCy) remain inconclusive. The present study aimed to thoroughly evaluate the effect of KIRs and binding ligands assessed by various models, in addition to other patient/donor variables, on clinical outcomes in haplo-HSCT. In a cohort of 354 patients undergoing their first haplo-HSCT, we found that a higher Count Functional inhibitory KIR score (CF-iKIR) was associated with improved progression-free survival (adjusted hazard ratio [HR], 0.71; *P* = .029) and overall survival (OS) (HR, 0.66; *P* = .016), while none of the other models predicted for survival in these patients. Moreover, using exploratory classification and regression tree analysis, we found that donor age <58 years combined with cytomegalovirus-nonreactive recipient was associated with the best OS, whereas donor age >58 years was associated with the worst OS. In the rest of our cohort (80%), cytomegalovirus-reactive recipients with a donor <58 years old, a higher CF-iKIR was associated with superior OS. The 3-year OS rates were 73.9%, 54.1% (HR, 1.84; *P* = .044), 44.5% (HR, 2.01; *P* = .003), and 18.5% (HR, 5.44; *P* <.001) in the best, better, poor, and worse donor groups, respectively. Our results suggest that KIR alloreactivity assessed by CF-iKIR score can help optimize donor selection in haplo-HSCT.

## Highlights

- Haplo-HSCT donor with NK cell alloreactivity predicted by count functional inhibitory KIR score is associated with improved survival
- A predictive algorithm incorporating NK cell alloreactivity and donor characteristics can aid haplo-HSCT donor selection

## Introduction

Allogeneic hematopoietic stem cell transplantation (HSCT) is the curative therapy for patients with advanced hematologic malignancies. T cell-mediated alloimmunity in allogeneic HSCT is associated with a beneficial graft-versus-leukemia (GVL) effect accompanied by an increased risk of graft-versus-host disease (GVHD). With the successful control of GVHD using prophylactic cyclophosphamide and graft engineering (1–3), in the past two decades, the use of haploidentical HSCT (haplo-HSCT) has rapidly expanded worldwide, and comparable outcomes have been observed between haplo-HSCT and HSCT with HLA-matched related or unrelated donors (2, 4–7). The use of haploidentical donors substantially expanded the availability of eligible donors, allowing a recipient to potentially have multiple donors. Therefore, donor selection and risk stratification are essential to achieve optimal outcomes.

Post-transplant cyclophosphamide (PTCy) has been widely used in haplo-HSCT to prevent GVHD mostly arising from the mismatched HLA haplotype. Moreover, robust immune reconstitution and preferential recovery of regulatory T cells (8) allow HSCT recipients who received PTCy to maintain a respectable anti-infection and antitumor immunity (9–11). Haploidentical donor selection criteria in the context of PTCy prophylaxis appear to be different from those used in other allogeneic HSCT platforms. Recent studies with haplo-HSCT using PTCy did not show a significant association between major clinical outcomes and the degree of HLA disparity assessed by cumulative mismatched alleles (12) or mismatched eplets (13). A registry-based study of HLA mismatches from individual locus showed that mismatches at certain loci are associated with favorable outcomes whereas other mismatches are deleterious (14), making it difficult to prioritize positive and negative selection factors that occur concomitantly in the same donor.

Natural killer (NK) cells have been hypothesized to contribute to the GVL effect without increasing GVHD, and the process is thought to be regulated by recognition between polymorphic killer cell immunoglobulin-like receptors (KIRs) and class-I HLA ligands expressed on the target cells (15). Pioneering studies in class-I HLA-mismatched HSCT showed that NK cell alloreactivity is associated with relapse protection and proposed a missing-self mechanism in which inhibitory KIRs on donor cells can no longer bind to their cognate ligands on the recipient cells (16, 17). With improved sequencing technology and understanding of the KIR genes and their expression, the receptor-ligand model was proposed to better predict NK cell alloreactivity based on the distinct combination of activating and inhibitory KIRs with their specific ligands. Improved survival and relapse protection were reportedly associated with the presence of activating KIR2DS1/HLA-C1C2 and reduced interaction between inhibitory KIR3DL1 and its HLA-Bw4 ligands (18, 19).

However, conflicting data emerged in registry-based studies of patients with acute myeloid leukemia (AML) (20) and patients with myelodysplastic syndrome (MDS) or secondary AML (21), illustrating the limitation of the current understanding of NK cell-mediated alloreactivity and the need for improvement in current KIR ligand modeling systems. Several novel models have been developed to better predict NK cell-mediated alloimmunity quantitatively by incorporating the missing inhibitory ligand component in addition to the activating KIR and inhibitory KIR contributions (22). Rather than focusing on the presence or absence of a particular receptor-ligand combination, Boelen et al. developed a functional inhibitory KIR scoring system that summarizes the functional engagement of inhibitory receptors and showed that an increased number of count functional inhibitory KIRs (CF-iKIR) score significantly enhanced CD8^+^ T cell survival and response against viral infections (23). In a comprehensive comparison study of the MDS/secondary AML cohort, while other KIR-ligand models failed to demonstrate the predictive value in a multivariable analysis, a higher CF-iKIR score was associated with superior event-free survival in patients who underwent HSCT from an unrelated donor (21). Whether this model is valid in haploidentical transplants is currently unknown.

PTCy administration transiently reduces mature NK cells (24) and may therefore weaken the effect of KIR-mediated NK cell alloimmunity in haplo-HSCT (25, 26). In the context of T cell-depleted haplo-HSCT, a beneficial effect of NK cell alloreactivity associated with reduced relapse rates was reported in several studies (17, 27). Investigations of KIR haplotype information showed that haplo-HSCT with KIR-B/X donors had a remarkedly reduced relapse rate compared with haplo-HSCT with KIR-A/A haplotype donors (28–30). Conflicting results were reported in T cell replete (TCR) haplo-HSCT, in which confounding adaptive immunity could affect KIR reconstitution (31) and obscure the anti-leukemia effect derived from NK cells (26, 30). Additionally, innate immunity recovers first after HSCT and acts as the first line of defense against foreign pathogens, and KIR ligand binding is essential in innate immunity against infections, akin to the anti-leukemia effect (32, 33).

In the present study, we aimed to comprehensively evaluate the effect of KIRs and their binding ligands along with other donor variables, on clinical outcomes in patients undergoing haplo-HSCT with PTCy-based GVHD prophylaxis.

## Methods

### Patient and transplant characteristics

Our cohort included 354 consecutively treated patients aged 18 years or older with hematologic malignancies who underwent their first unmanipulated haplo-HSCT at The University of Texas MD Anderson Cancer Center (MDACC) between May 2009 and September 2019. All patients received PTCy-based GVHD prophylaxis with tacrolimus and mycophenolate, as previously described by our group (34). Patients with a high level (mean fluorescence intensity >2,000) of donor-specific anti-HLA antibodies received desensitization therapy before HSCT per institutional protocol (35). Comorbidities before HSCT were evaluated using the Hematopoietic Cell Transplant-Comorbidity Index (HCT-CI) (36) and hematologic malignancies were risk-stratified using the refined Disease Risk Index (DRI) (37). Clinical and laboratory data were collected from electronic medical records. The study protocol was approved by the Institutional Review Board of MDACC. The ethics committee waived the requirement of written informed consent for participation.

### HLA genotyping and KIR genotyping

Patients eligible for the study had donor and recipient HLA typing performed at the HLA-A, -B, -C, -DRB1, -DQB1 and -DPB1 loci using sequence-based typing methods at high resolution (38). KIR genotyping was performed by KIR sequence-specific oligonucleotide probes (Thermo Fisher Scientific Life Science, Waltham, MA; and One Lambda, Canoga Park, CA).

### KIR haplotype assignment and KIR ligand– and KIR motif-based classification models

We examined and compared the following models to assess NK cell alloreactivity: donor NK cell benefit, KIR2DS1/C1C2 epitope combination, donor centromeric motif, donor telomeric motif, KIR B-content score, inhibitory KIR score, and CF-iKIR score.

For the donor NK cell benefit model, the NK cell alloreactivity was predicted based on high-resolution HLA typing of the donor and recipient, as described previously (39). Briefly, KIR ligand HLA-C and HLA-B molecules were grouped into three major categories (C1, C2, Bw4) based on the specific amino acid sequence that defines specific KIR ligand binding https://www.ebi.ac.uk/ipd/kir/ligand.html. NK cell alloreactivity in the graft-versus-host direction was assigned when the recipient lacked at least one of the HLA ligands that were present in the donor. For the KIR2DS1/C1C2 epitope combination model, binding between the KIR2DS1 and C1C2 ligands was classified as described by Venstrom et al. (19) For the donor centromeric motif and telomeric motif models, donor A or B haplotypes were assigned according to the definition described by Cooley et al, based on the presence or absence of KIR-B–specific genes (40, 41). For the KIR B-content score model, donors were classified into three groups (neutral, better, best) using the B-content score and the presence of the Cen-B/B motif, as described previously (41).

For the inhibitory KIR score and CF-iKIR score models, as described by Schetelig et al. (21) and Boelen et al (23), the inhibitory score was calculated based on the donor’s KIR genotype and the recipient’s HLA ligands, and KIR was considered functional only when the cognate ligands were exhibited by the recipient’s HLA molecules. Thus, inhibitory score = 1 if functional KIR2DL1 + 1 if strong functional KIR2DL2 or 0.5 if weak functional KIR2DL2 + 0.75 if functional KIR2DL3 + 1 if functional KIR3DL1. Similarly, the CF-iKIR score was calculated based on the donor’s KIR genotype and the recipient’s HLA ligands: CF-iKIR score = 1 if functional KIR2DL1 + 1 if functional KIR2DL2 and/or functional KIR2DL3 + 1 if functional 3DL1 (23).

### Clinical endpoints and statistical methods

Baseline patient and HSCT-related factors and NK cell alloreactivity as assessed by the various models were summarized using descriptive statistics. The primary outcome was overall survival (OS), and secondary outcomes included progression-free survival (PFS), relapse, non-relapse mortality (NRM), acute GVHD (aGVHD), chronic GVHD (cGVHD), and viral reactivation. All outcomes were measured from the time of stem cell infusion. OS was defined as the time from stem cell infusion to death from any cause. PFS events included death or relapse. NRM was defined as death without a previous relapse. Patients without the event were censored at the time of the last contact. The Kaplan-Meier method was used to calculate unadjusted PFS and OS, and the cumulative incidence with competing risks method was used to calculate aGVHD, cGVHD, NRM, relapse, and viral reactivation. For NRM, relapse was the competing risk, and for relapse, the competing risk was NRM. For aGVHD and cGVHD, death without the event and relapse were the competing risks. The effects of NK cell alloreactivity as assessed by various models and the effects of baseline clinical factors on survival outcomes were analyzed using univariate and multivariable Cox proportional hazards regression models, and the effects of these factors on relapse, NRM, aGVHD, cGVHD and viral reactivation were determined by univariate and multivariable proportional subdistribution hazards regression models. The median cutoff of the inhibitory KIR score and CF-iKIR score was used in the analyses due to it provided the lowest Akaike, Bayesian information criterion, as well as maximum log-likelihood in a univariate regression model for each outcome of interest.

Baseline clinical factors included in the univariate models were recipient age (continuous), sex, recipient-donor sex combination (female donor to male recipient vs. others), hematopoietic cell transplant comorbidity index (HCT-CI), transplant protocol (on protocol vs. standard of care), donor age (continuous), ABO matching (match vs. mismatches), recipient-donor CMV serostatus (nonreactive-nonreactive vs. nonreactive-reactive vs. reactive-nonreactive vs. reactive-reactive), stem cell type (peripheral blood vs. bone marrow), conditioning regimen intensity (myeloablative vs. reduced intensity/non-myeloablative), donor-specific anti-HLA antibodies (presence vs. absence).

Variables with a P-value <0.10 in the univariate analysis were included in the multivariable analysis. All variables of interest were tested for the proportional hazards assumption and interaction terms. All tests were two-sided. The type 1 error rate was fixed at 0.05. No adjustments for multiple testing were made.

Classification and regression tree analysis (42) of OS was used to develop an algorithm for donor selection by incorporating donor characteristics that significantly predicted OS in univariate analyses with adjustment for significant recipient characteristics. The donor selection algorithm was validated in a new dataset created using the bootstrapping method.

Stata statistical software (SE 13, StataCorp LP, College Station, TX) was used for statistical analyses.

## Results

### Patient characteristics and KIR alloreactivity models

The analysis included 354 patients with a median age of 49 years (range 18-72 years), of which 84 (23.7%) were ≥60 years. Patient and HSCT-related characteristics, as well as NK cell alloreactivity predicted by various models, are listed in **Table 1**. The diagnosis was AML/MDS in 204 patients (57.6%), acute lymphoblastic leukemia in 59 patients (16.7%), and others (chronic lymphoblastic leukemia/myeloproliferative neoplasm, Hodgkin or non-Hodgkin lymphoma, chronic lymphoblastic leukemia, or myeloma) in 91 patients (25.7%). One hundred forty-four patients (40.7%) had a high DRI and 25 (7.1%) had a very high DRI. Fifty-one percent of the patients had an HCT-CI ≥3. Myeloablative conditioning was used in 200 patients (56.5%), and most patients (84.5%) received a bone marrow graft. Donor-specific anti-HLA antibodies were identified in 33 patients (9.3%). Cytomegalovirus (CMV) serostatus was nonreactive in 48 recipients (13.6%) and 142 donors (40.1%).

**Table 1:** Patient and HSCT-related characteristics and NK cell alloreactivity in our cohort of patients who underwent haploidentical HSCT (n = 354).

Variable	No. (%)
Male	209 (59.0)
Female donor to male recipient	69 (19.5)
Median recipient age (range)	49 years (18-72 years)
Age ≥60 years	84 (23.7)
Median HCT-CI (range)	3 (0-12)
Diagnosis	
AML/MDS	204 (57.6)
ALL	59 (16.7)
CML/MPN	39 (11.0)
NHL	27 (7.6)
Hodgkin lymphoma	12 (3.4)
CLL	11 (3.1)
Myeloma	2 (0.6)
Disease risk index	
Low	33 (9.3)
Intermediate	152 (42.9)
High	144 (40.7)
Very high	25 (7.1)
Median donor age (range)	34 years (11-67 years)
Donor age >40 years	123 (34.7)
ABO match	
Match	241 (68.1)
Minor mismatch	59 (16.7)
Major mismatch	51 (14.4)
Bidirectional mismatch	3 (0.8)
Recipient CMV-reactive	304 (85.9)
Recipient-donor CMV serostatus	
NR-NR	30 (8.5)
NR-R	18 (5.1)
R-NR	112 (31.6)
R-R	192 (54.2)
Missing	2 (0.5)
Stem cell source	
Bone marrow	299 (84.5)
Peripheral blood	55 (15.5)
Conditioning intensity	
MAC	200 (56.5)
RIC/NMA	154 (43.5)
Donor-specific anti-HLA antibodies (MFI>1,000)	33 (9.3)
Donor relationship	
Child	178 (50.3)
Sibling	136 (38.4)
Parent and other (other donors, n=3)	40 (11.3)
NK cell alloreactivity model	
Donor NK cell benefit	
No	253 (71.5)
Yes	101 (28.5)
KIR2DS1/C1C2 epitope combination	
Negative	222 (62.7)
Positive: recipient C1+	115 (32.5)
Positive: recipient C2/C2	17 (4.8)
Donor centromeric motif	
A/A	182 (51.4)
A/B	135 (38.1)
B/B	37 (10.5)
Donor telomeric motif	
A/A	232 (65.5)
A/B	106 (29.9)
B/B	16 (4.5)
KIR B-content score	
Neutral	263 (74.3)
Better	54 (15.3)
Best	37 (10.5)
Inhibitory KIR score	
Mean (SD)	2.29 (0.86)
Median (range)	2.5 (0.5-3.75)
>2.5	159 (44.9)
Count functional inhibitory KIR score	
Mean (SD)	2.12 (0.73)
Median (range)	2 (0-3)
>2	116 (32.8)

HSCT, hematopoietic stem cell transplant; NK cell, natural killer cell; HCT-CI, hematopoietic cell transplant-comorbidity index; AML, acute myeloid leukemia; MDS, myelodysplastic syndrome; ALL, acute lymphoblastic leukemia; CML, chronic myeloid leukemia; MPN, myeloproliferative neoplasm; NHL, non-Hodgkin lymphoma; CLL, chronic lymphoblastic leukemia; CMV, cytomegalovirus; NR, nonreactive; R, reactive; MAC, myeloablative conditioning; RIC, reduced-intensity conditioning; NMA, nonmyeloablative; HLA, human leukocyte antigen; KIR, killer cell immunoglobulin-like receptor.

**Figure 1:**
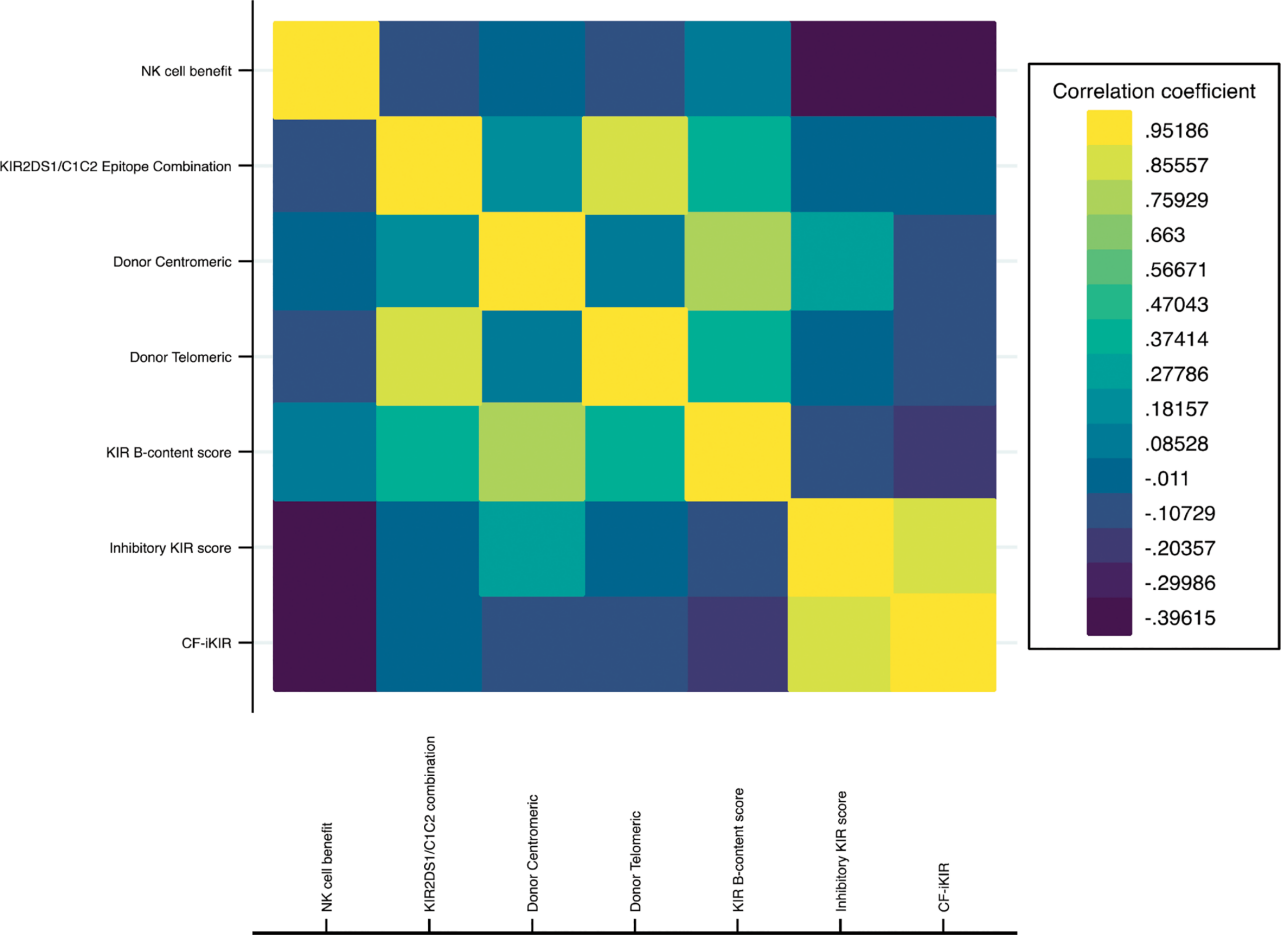
Correlation heatmap of NK cell alloreactivity models. Abbreviations: NK cell, natural killer cell; KIR, killer cell immunoglobulin-like receptor; CF-iKIR score, count functional inhibitory KIR score.

Based on HLA typing from donor and recipient pairs, the NK cell benefit occurred in 28.5% of our cohort. For the KIR B-content score derived from the donor KIR genotype, donors were categorized as neutral (74.3%), better (15.3%), or best (10.5%) based on the number of centromeric and telomeric B motifs. The inhibitory KIR score and CF-iKIR score, calculated based on the presence of donor inhibitory KIRs and cognate HLA ligands from the recipient, had mean values of 2.29 for the inhibitory KIR score and 2.12 for the CF-iKIR score. Not surprisingly, the neutral/better/best classification for B-content score partially correlated with donor centromeric motif and donor telomeric motif. Inhibitory KIR scores largely overlapped with CF-iKIR scores but with a higher median cutoff (**Figure 1** and **Supplemental Table 1**).

### Effect of NK cell alloreactivity on clinical outcomes by different models

**Figure 2:**
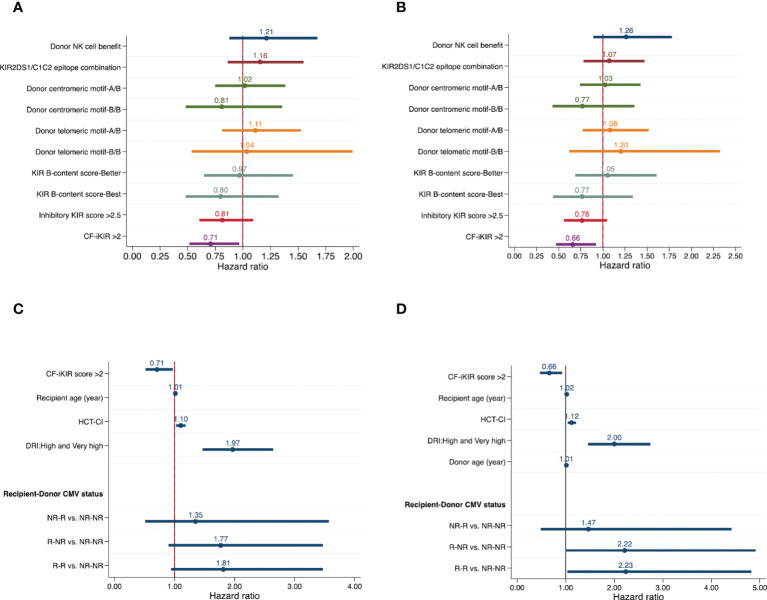
Effects of NK cell alloreactivity according to various models and patient and transplant-related factors on survival outcomes of patients who underwent haploidentical hematopoietic stem cell transplant (n = 354). Forest plots show effects of NK cell alloreactivity on progression-free survival **(A)** and overall survival **(B)** and effects of patient- and transplant-related factors on progression-free survival **(C)** and overall survival **(D)**, after adjustment for other significant baseline clinical factors. Abbreviations: NK, natural killer cells; KIR, killer cell immunoglobulin-like receptor; HCT-CI, hematopoietic cell transplant-comorbidity index; DRI, disease risk index; CMV, cytomegalovirus; NR, nonreactive; R, reactive.

Unadjusted effects of NK cell alloreactivity according to various models on HSCT outcomes are summarized in **Table 2**. Only the CF-iKIR score model was associated with post-HSCT survival; the other models failed to predict survival outcomes. Using the median CF-iKIR score as a cutoff, univariate analysis showed that the 3-year PFS estimate for patients with low CF-iKIR scores was 39.4%, and for those with high CF-iKIR scores, 50.7%; 3-year OS estimates were 45.0% for low CF-iKIR score and 55.1% for high CF-iKIR score. The effects of CF-iKIR score on survival persisted after adjustment for other significant baseline clinical factors, with a hazard ratio (HR) of 0.71 (95% confidence interval [CI], 0.52-0.96; *P* = .029) for PFS (**Figure 2A**) and 0.66 (95% CI, 0.48-0.93; *P* = .016) for OS (**Figure 2B**).

**Table 2:** Univariate analysis of the effect of NK cell alloreactivity predicted by different models on haploidentical HSCT outcomes in our cohort (n = 354).

NK cell alloreactivity model	PFS			OS			Relapse			NRM			aGVHD grade 2-4			cGVHD			Viral reactivation		
	At 3 years	HR (95% CI)	P	At 3 years	HR (95% CI)	P	At 3 years (95% CI)	HR (95% CI)	P	At 3 years	HR (95% CI)	P	At 100 days	HR (95% CI)	P	At 3 years	HR (95% CI)	P	At 100 days	HR (95% CI)	P
Donor NK cell benefit																					
No	45.23 (38.60-51.62)	Ref	Ref	49.53 (42.54-56.13)	Ref	Ref	22.79 (17.65-28.34)	Ref	Ref	31.98 (26.02-38.07)	Ref	Ref	31.22 (25.61-36.98)	Ref	Ref	13.64 (9.54-18.47)	Ref	Ref	68.7 (62.6-74.0)	Ref	
Yes	37.30 (27.08-47.49)	1.15 (0.84-1.57)	0.379	45.05 (34.13-55.34)	1.14 (0.82-1.58)	0.443	31.93 (22.34-41.91)	1.36 (0.87-2.14)	0.18	30.77 (21.70-40.29)	0.92 (0.60-1.40)	0.692	39.60 (30.08-48.95)	1.32 (0.91-1.92)	0.147	11.70 (6.00-19.49)	0.90 (0.45-1.77)	0.758	82.2 (73.2-88.4)	1.31 (1.04-1.66)	0.024
KIR2DS1/C1C2 epitope combination*																					
Negative	45.75 (38.59-52.61)	Ref	Ref	50.09 (42.54-57.16)	Ref	Ref	23.04 (17.47-29.08)	Ref	Ref	31.21 (24.91-37.70)	Ref	Ref	32.43 (26.79-39.09)	Ref	Ref	11.44 (7.50-16.28)	Ref	Ref	72.6 (66.2-78.0)	Ref	
Positive	38.57 (29-68-47.36)	1.19 (9.90-1.59)	0.227	45.03 (35.54-54.06)	1.16 (0.85-1.58)	0.342	29.04 (21.19-37.34)	1.28 (0.83-1.98)	0.261	32.39 (24.21-40.82)	1.03 (0.70-1.52)	0.892	34.48 (26.84-42.96)	1.14 (0.79-1.65)	0.493	16.14 (10.02-23.55)	1.29 (0.70-2.36)	0.412	72.5 (64.0-79.4)	0.99 (0.78-1.27)	0.972
Donor centromeric motif																					
A/A	43.90 (36.21-51.32)	Ref	Ref	50.31 (42.36-57.72)	Ref	Ref	27.24 (20.71-34.16)	Ref	Ref	28.86 (22.26-35.76)	Ref	Ref	37.36 (30.37-44.34)	Ref	Ref	15.53 (10.41-21.59)	Ref	Ref	74.9 (67.9-80.6)	Ref	
A/B	40.79 (31.73-49.62)	1.06 (0.79-1.42)	0.712	44.03 (34.29-53.32)	1.08 (0.79-1.48)	0.624	22.71 (15-72-30.50)	0.80 (0.51-1.27)	0.345	36.50 (27.93-45.09)	1.25 (0.85-1.85)	0.256	27.41 (20.19-35.99)	0.73 (0.48-1.09)	0.124	9.38 (4.96-15.49)	0.56 (0.28-1.13)	0.103	68.9 (60.4-76.0)	0.86 (0.67-1.12)	0.266
B/B	47.93 (29.75-64.02)	0.79 (0.73-1.32)	0.375	55.62 (36.17-71.28)	0.75 (0.43-1.32)	0.32	24.48 (11.44-40.10)	0.85 90.40-1.80)	0.67	27.58 (13.57-43.58)	0.82 90.41-1.64)	0.567	37.84 (22.62-52.97)	1.05 (0.59-1.86)	0.869	15.76 (5.74-30.26)	0.98 (0.38-2.55)	0.975	75.0 (57.5-86.2)	0.92 (0.63-1.37)	0.696
Donor telomeric motif																					
A/A	44.70 (37.72-51.42)	Ref	Ref	49.76 (42.40-56.69)	Ref	Ref	23.94 (18.39-29.90)	Ref	Ref	31.36 (25.17-37.72)	Ref	Ref	33.62 (27.62-39.72)	Ref	Ref	11.49 (7.60-16.32)	Ref	Ref	73.2 (67.0-78.5)	Ref	
A/B	42.53 (32.46-52.22)	1.11 (0.81-1.52)	0.505	49.00 (38.30-58.84)	1.10 (0.78-1.53)	0.589	27.97 (19.37-37.20)	1.18 (0.74-1.88)	0.486	29.50 (20.91-38.58)	0.97 (0.63-1.49)	0.899	32.08 (23.43-41.02)	0.96 (0.64-1.43)	0.833	15.71 (9.07-24.01)	1.25 (0.65-2.39)	0.497	72.6 (63.1-80.1)	1.02 (0.78-1.32)	0.888
B/B	25.31 (6.74-49.67)	1.31 (0.71-2.42)	0.394	24.06 (6-16-48.28)	1.60 (0.86-2.97)	0.139	25.78 (7.93-48.42)	1.06 (0.39-2.89)	0.904	48.90 (21.86-71.43)	1.45 (0.70-3.00)	0.323	43.75 (19.81-65.56)	1.36 (0.65-2.88)	0.416	20.54 (5.04-43.24)	1.78 (0.54-5.83)	0.339	62.5 (34.9-81.1)	0.66 (0.38-1.13)	0.128
KIR B-content score																					
Neutral	43.12 (36.67-49.40)	Ref	Ref	48.88 (42.90-55.32)	Ref	Ref	26.30 (20.89-32.02)	Ref	Ref	30.57 (24.86-36.46)	Ref	Ref	33.84 (28.18-39.57)	Ref	Ref	13.28 (9.29-17.99)	Ref	Ref	72.4 (66.5-77.4)	Ref	
Better	40.14 (26.26-53.63)	1.02 (0.69-1.52)	0.911	41.47 (26.90-55.42)	1.13 (0.75-1.70_	0.556	20.54 (10.89-32.77)	0.71 (0.37-1.37)	0.304	39.32 (25.79-52.57)	1.28 (0.78-2.11)	0.331	29.63 (18.17-41.99)	0.90 (0.51-1.56)	0.71	11.21 (4.10-22.31)	0.75 (0.30-1.91)	0.549	72.2 (58.2-82.2)	1.00 (0.71-1.41)	0.997
Best	47.93 (29.75-64.02)	0.78 (0.47-1.28)	0.324	55.62 (36.17-71.28)	0.74 (0.42-1.29)	0.289	24.48 (11.45-40.10)	0.88 (0.42-1.84)	0.737	27.58 (13.57-43.58)	0.77 (0.39-1.52)	0.457	37.84 (22.62-52.97)	1.17 (0.67-2.05)	0.587	15.75 (5.74-30.26)	1.17 (0.46-2.98)	0.749	75.0 (57.7-86.2)	0.98 (0.67-1.44)	0.929
Inhibitory KIR score**																					
Continuous	NA	0.93 (0.80-1.11)	0.456	NA	0.94 (0.79-1.11)	0.468	NA	8.39 (0.65-1.09)	0.185	NA	1.06 (0.87-1.30)	0.566	NA	0.79 (0.64-0.99)	0.04	NA	0.99 (0.72-1.36)	0.956		0.93 (0.81-1.06)	0.276
≤2.5	39.48 (32.09-46.76)	Ref	Ref	45.89 (38.20-53.23)	Ref	Ref	28.87 (22.31-35.74)	Ref	Ref	31.65 (29.97-38.53)	Ref	Ref	37.43 (30.67-44.18)	Ref	Ref	11.25 (7.04-16.53)	Ref	Ref	77.9 (69.2-81.3)	Ref	
>2.5	47.46 (38.97-55.48)	0.83 (0.62-1.10)	0.196	51.14 (41.99-59.56)	0.82 (0.60-1.11)	0.191	20.87 (14.73-27.76)	0.73 (0.47-1.13)	0.16	31.66 (24.17-39.40)	1.00 (0.69-1.45)	0.997	28.93 (22.10-36.09)	0.70 (0.49-0.99)	0.048	15.52 (10.09-22.02)	1.37 (0.75-2.50)	0.305	68.6 (60.8-75.2)	0.86 (0.68-1.09)	0.222
CF-iKIR score**																					
Continuous	NA	0.90 (0.74-1.08)	0.246	NA	0.89 (0.73-1.08)	0.246	NA	0.88 (0.67-1.17)	0.388	NA	0.97 (0.76-1.23)	0.793	NA	0.82 (0.64-1.05)	0.113	NA	1.25 (0.76-2.05)	0.376		0.95 (0.80-1.11)	0.495
≤2	39.36 (32.69-45.94)	Ref		45.00 (38.05-51.69)	Ref	Ref	27.23 (21.42-33.34)	Ref	Ref	33.41 (27.24-39.69)	Ref	Ref	35.29 (29.27-41.36)	Ref	Ref	10.64 (6.91-15.30)	Ref	Ref	73.9 (67.8-79.0)	Ref	
>2	50.69 (40.55-59.97)	0.73 (0.54-0.99)	0.048	55.12 (44.20-64.76)	0.70 (0.50-0.97)	0.032	21.28 (14.10-29.45)	0.8 (0.51-1.27)	0.347	28.03 (19.68-36.96)	0.81 (0.54-1.21)	0.299	30.17 (22.10-38.63)	0.84 (0.56-1.24)	0.376	18.33 (11.52-26.41)	1.73 (0.95-3.17)	0.075	69.9 (60.6-77.4)	0.91 (0.70-1.17)	0.472

NK cell, natural killer cell; HSCT, hematopoietic stem cell transplant; PFS, progression-free survival; OS, overall survival; NRM, non-relapse mortality; aGVHD, acute graft-versus-host disease; cGVHD, chronic graft-versus-host disease; HR, hazard ratio; CI, confidence interval; KIR, killer cell immunoglobulin-like receptor; CF-iKIR score, count functional inhibitory KIR score.

*Due to only 17 patients (4.8%) were in positive: recipient C2/C2 group, this group was combined with positive: recipient C1+.

**The median cutoff was used in the analysis due to it provided the lowest AIC, BIC and maximum log-likelihood.

While the other models failed to predict survival they did show correlations with other transplant outcomes. A high inhibitory KIR score predicted a reduced risk of clinically significant aGVHD (grade 2-4), with a cumulative incidence at 100 days of 37.4% for low inhibitory KIR score and 28.9% for high inhibitory KIR score (adjusted HR, 0.66; 95% CI, 0.46-0.93; *P* = .02). The risk of viral reactivation significantly increased in patients who received a stem cell graft from a donor with NK cell alloreactivity predicted by the NK cell benefit model (adjusted HR, 1.30; 95% CI, 1.02-1.66; *P* = .033). None of the NK cell alloreactivity models predicted relapse, NRM, or cGVHD after HSCT. Results of multivariable analyses of the effect of NK cell alloreactivity according to various models on HSCT outcomes are shown in **Table 3**.

**Table 3:** Multivariable analysis of the effect of NK cell alloreactivity predicted by different models on haploidentical HSCT outcomes in our cohort (n = 354).

NK cell alloreactivity model	PFS			OS			Relapse			NRM			aGVHD grade 2-4			cGVHD			Viral reactivation		
	Adj. HR	95% CI	P	Adj. HR	95% CI	P	Adj. HR	95% CI	P	Adj. HR	95% CI	P	Adj. HR	95% CI	P	Adj. HR	95% CI	P	Adj. HR	95% CI	P
Donor NK cell benefit																					
No	Ref			Ref			Ref			Ref			Ref			Ref			Ref		
Yes	1.21	0.88-1.67	0.239	1.26	0.89-1.78	0.185	1.31	0.83-2.07	0.24	1.13	0.74-1.72	0.563	1.40	0.96-2.03	0.076	0.90	0.46-1.76	0.761	1.30	1.02-1.66	0.033
KIR2DS1/C1C2 epitope combination*																					
Negative	Ref			Ref			Ref			Ref			Ref			Ref			Ref		
Positive	1.15	0.86-1.54	0.332	1.07	0.78-1.47	0.663	1.42	0.90-2.24	0.132	0.95	0.64-1.41	0.809	1.13	0.78-1.63	0.518	1.30	0.72-2.38	0.387	1.03	0.81-1.31	0.822
Donor centromeric motif																					
A/A	Ref			Ref			Ref			Ref			Ref			Ref			Ref		
A/B	1.02	0.75-1.38	0.91	1.03	0.74-1.42	0.867	0.82	0.52-1.31	0.42	1.12	0.74-1.69	0.604	0.71	0.47-1.06	0.095	0.57	0.28-1.17	0.124	0.82	0.64-1.07	0.14
B/B	0.81	0.48-1.35	0.42	0.77	0.43-1.35	0.361	0.83	0.39-1.75	0.627	0.88	0.46-1.68	0.694	1.06	0.60-1.87	0.833	0.97	0.38-2.48	0.957	1.02	0.69-1.51	0.918
Donor telomeric motif																					
A/A	Ref			Ref			Ref			Ref			Ref			Ref			Ref		
A/B	1.11	0.81-1.52	0.506	1.08	0.77-1.52	0.645	1.23	0.75-2.00	0.408	0.98	0.63-1.52	0.936	0.98	0.66-1.47	0.929	1.29	0.68-2.48	0.432	1.04	0.81-1.40	0.752
B/B	1.04	0.54-1.99	0.916	1.2	0.62-2.32	0.578	1.17	0.41-3.33	0.767	1.17	0.56-2.46	0.679	1.26	0.59-2.68	0.547	1.91	0.62-5.88	0.262	0.61	0.34-1.10	0.102
KIR B-content score																					
Neutral	Ref			Ref			Ref			Ref			Ref			Ref			Ref		
Better	0.97	0.65-1.45	0.883	1.05	0.69-1.60	0.807	0.79	0.40-1.57	0.513	1.16	0.70-1.92	0.572	0.88	0.51-1.52	0.64	0.76	0.30-1.91	0.561	0.99	0.69-1.40	0.937
Best	0.80	0.48-1.32	0.384	0.77	0.44-1.34	0.347	0.87	0.42-1.80	0.707	0.86	0.46-1.62	0.641	1.19	0.68-2.06	0.547	1.14	0.45-2.86	0.781	1.10	0.75-1.62	0.619
Inhibitory KIR score, continuous	0.94	0.79-1.11	0.47	0.92	0.77-1.10	0.346	0.90	0.69-1.17	0.413	0.94	0.75-1.18	0.584	0.78	0.612-0.97	0.027	1.02	0.76-1.38	0.882	0.89	0.79-1.03	0.124
Inhibitory KIR score																					
≤2.5	Ref			Ref			Ref			Ref			Ref			Ref			Ref		
>2.5	0.81	0.61-1.09	0.166	0.76	0.56-1.05	0.092	0.79	0.51-1.22	0.284	0.83	0.56-1.23	0.364	0.66	0.46-0.93	0.02	1.46	0.80-2.66	0.219	0.82	0.64-1.94	0.099
CF-iKIR score, continuous	0.90	0.74-1.10	0.303	0.88	0.72-1.08	0.223	0.95	0.70-1.27	0.718	0.86	0.66-1.11	0.24	0.81	0.63-1.04	0.093	1.29	0.79-2.08	0.306	0.93	0.7901.09	0.345
CF-iKIR score																					
≤2	Ref			Ref			Ref			Ref			Ref			Ref			Ref		
>2	0.71	0.52-0.96	0.029	0.66	0.48-0.93	0.016	0.94	0.59-1.51	0.798	0.69	0.45-1.04	0.079	0.83	0.56-1.24	0.365	1.77	0.96-3.26	0.067	0.89	0.69-1.15	0.373

NK cell, natural killer cell; HSCT, hematopoietic stem cell transplant; PFS, progression-free survival; OS, overall survival; NRM, non-relapse mortality; aGVHD, acute graft-versus-host disease; cGVHD, chronic graft-versus-host disease; HR, hazard ratio; CI, confidence interval; KIR, killer cell immunoglobulin-like receptor; CF-iKIR score, count functional inhibitory KIR score.

*Due to only 17 patients (4.8%) were in positive: recipient C2/C2 group, this group was combined with positive: recipient C1+.

### Effect of recipient, donor, and HSCT-related characteristics on clinical outcomes

Results of univariate analyses of the effect of baseline clinical characteristics on HSCT outcomes are shown in **Supplemental Table 2**. After adjustment for all significant factors identified in univariate models, we found that older recipient age (HR, 1.01; 95% CI, 1.00-1.02; *P* = .011), higher HCT-CI (HR, 1.10; 95% CI, 1.03-1.18; *P* = .004), and high/very high DRI (HR, 1.97; 95% CI, 1.47-2.64; *P* <.001) were clinical factors independently associated with poor PFS (**Figure 2C**).

For OS, older recipient age (HR, 1.02; 95% CI, 1.00-1.03; *P* <.001), higher HCT-CI (HR, 1.12; 95% CI, 1.04-1.20; *P* = .002), high or very high DRI (HR, 2.00; 95% CI, 1.46-2.74; *P* <.001), recipient-donor CMV serostatus (HR, 2.22; 95% CI, 1.00-4.91; *P* = .041) for reactive recipient–nonreactive donor and (HR, 2.23; 95% CI, 1.03-4.82; *P* = .024) for reactive recipient–reactive donor when compared with nonreactive recipient–nonreactive donor), and older donor (HR, 1.01; 95% CI, 1.00-1.03; *P* = .043) were predictors of poor OS (**Figure 2D**).

Independent predictors of an increased risk of NRM were older recipient age (HR, 1.03; 95% CI, 1.01-1.04; *P* <.001), higher HCT-CI (HR, 1.12; 95% CI, 1.02-1.22; *P* = .016), and older donor age (HR, 1.02; 95% CI, 1.00-1.04; *P* = .011). We also found a significant interaction between donor age and donor sex on the risk of NRM. Using a stem cell graft from a female donor aged 40 years or older was associated with a significantly increased risk of NRM compared with a male donor younger than 40 years, with an adjusted HR of 2.00 (95% CI, 1.15-3.46; *P* = .014). The increased NRM associated with an older female donor likely resulted from the development of severe aGVHD as we also found a significantly increased risk of grade 3-4 aGVHD associated with using a stem cell graft from a female donor aged 40 years or older compared with a male donor younger than 40 years (HR, 4.09; 95% CI, 1.48-11.34; *P* = .007; **Supplemental Table 3**). Other clinical factors predicting increased risk of grade 3-4 aGVHD were the use of a stem cell graft from a female donor to a male recipient and the recipient having donor-specific anti-HLA antibodies. Higher HCT-CI was associated with an increased risk of grade 2-4 aGVHD. High/very high DRI was the only factor associated with an increased risk of relapse (HR, 2.48; 95% CI, 1.60-3.86; *P* <.001). Results of multivariable analyses of the effect of clinical factors on post-HSCT outcomes are detailed in **Table 4**.

**Table 4:** Multivariable analyses of the effect of baseline clinical characteristics on outcomes of patients who underwent haploidentical hematopoietic stem cell transplant (n = 354).

	Adjusted HR*	95%CI	P
**Model 1. PFS**			
Recipient age, continuous	1.01	1.00-1.02	0.011
HCT-CI, continuous	1.10	1.03-1.18	0.004
Disease risk index			
Low/intermediate	Ref		
High/very high	1.97	1.47-2.64	<0.001
Recipient-donor CMV status			
NR-NR	Ref		
NR-R	1.35	0.51-3.57	0.544
R-NR	1.77	0.90-3.47	0.096
R-R	0.81	0.95-3.47	0.073
**Model 2. OS**			
Recipient age, continuous	1.02	1.00-1.03	<0.001
HCT-CI, continuous	1.12	1.04-1.20	0.002
Disease risk index			
Low/intermediate	Ref		
High/very high	2.00	1.46-2.74	<0.001
Recipient-donor CMV status			
NR-NR	Ref		
NR-R	1.47	0.48-4.41	0.386
R-NR	2.22	1.00-4.91	0.041
R-R	2.23	1.03-4.82	0.024
Donor age, continuous	1.01	1.00-1.03	0.043
**Model 3. Relapse**			
Recipient age, continuous	0.99	0.97-1.00	0.07
Disease risk index			
Low/intermediate	Ref		
High/very high	2.48	1.60-3.86	<0.001
Donor age, continuous	0.98	0.97-1.00	0.091
**Model 4. NRM**			
Recipient age, continuous	1.03	1.01-1.04	<0.001
HCT-CI, continuous	1.12	1.02-1.22	0.016
Donor age, continuous	1.02	1.00-1.04	0.011
Recipient-donor CMV status			
NR-NR	Ref		
NR-R	0.84	0.22-3.24	0.795
R-NR	1.59	0.64-3.94	0.315
R-R	2.16	0.91-5.15	0.082
**Model 5. aGVHD grade 2-4**			
HCT-CI, continuous	1.09	1.00-1.19	0.044
Conditioning regimen intensity			
MAC	Ref		
RIC/NMA	1.39	0.98-1.99	0.065
Stem cell type			
Bone marrow	Ref		
Peripheral blood	0.8	0.50-1.45	0.561
**Model 6. aGVHD grade 3-4**			
Sex mismatch			
Other combination	Ref		
Female donor to male recipient	2.74	1.20-6.24	0.016
Donor age, continuous	1.04	1.01-1.08	0.013
Donor specific anti-HLA antibodies	4.45	1.54-12.88	0.006
**Model 7. cGVHD**			
Donor relationship			
Child	Ref		
Sibling	0.99		
Parent or other family donor	2.14	0.96-4.75	0.06

PFS, progression-free survival; OS, overall survival; NRM, non-relapse mortality; aGVHD, acute graft versus host disease; cGVHD, chronic graft versus host disease; HR, hazard ratio; CI, confidence interval; HCT-CI, hematopoietic cell transplant comorbidity index; CMV, cytomegalovirus; NR, nonreactive; R, reactive; MAC, myeloablative conditioning; RIC, reduced-intensity conditioning; NMA, nonmyeloablative.

*All models were adjusted for the impact of NK cell alloreactivity predicted by the CF-iKIR score.

### Donor selection algorithm based on the CF-iKIR score and donor characteristics

**Figure 3:**
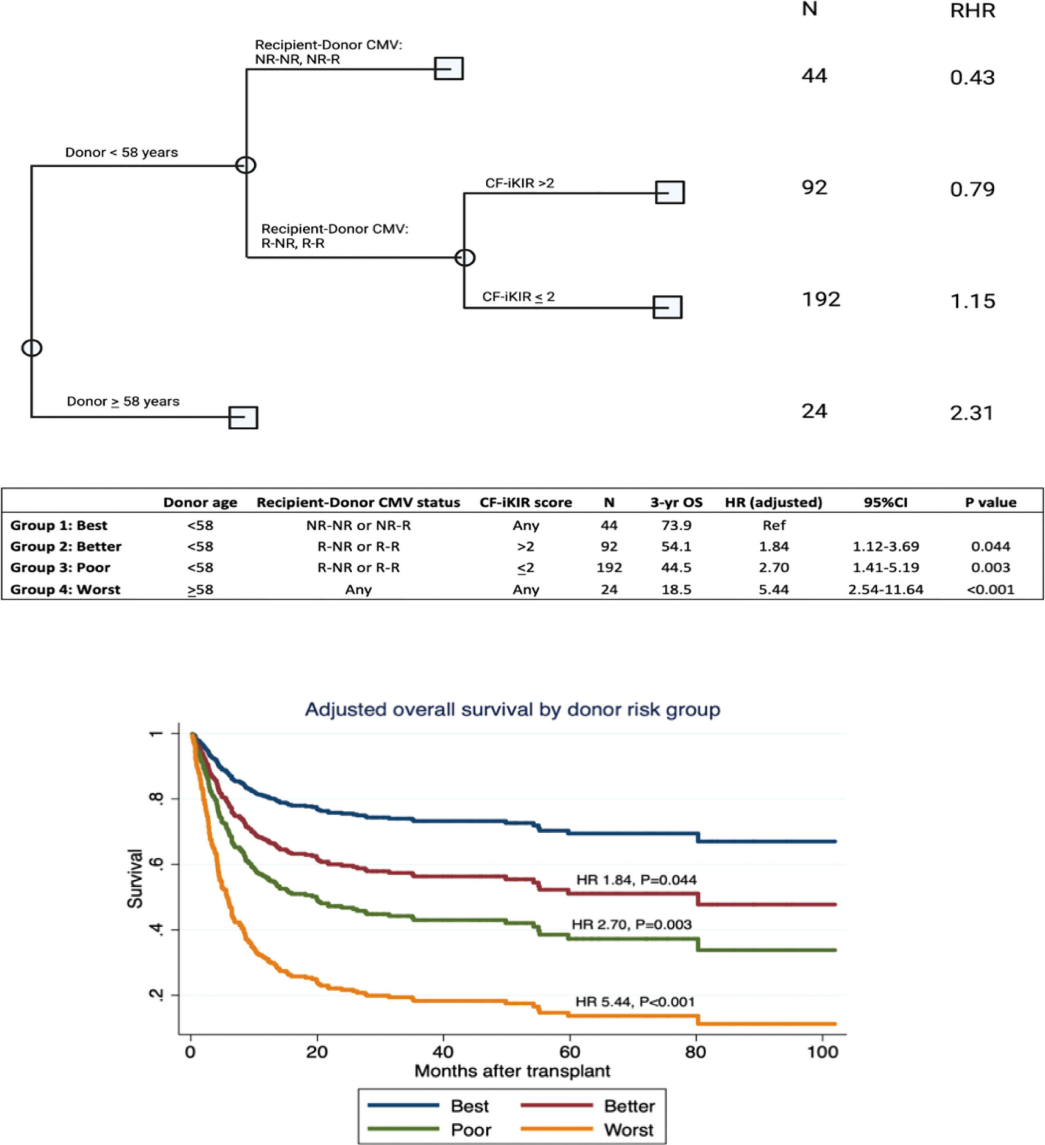
Algorithm for donor selection based on donor characteristics and natural killer cell alloreactivity predicted by CF-iKIR score. Abbreviations: CF-iKIR score, count functional inhibitory killer cell immunoglobulin-like receptor score; CMV, cytomegalovirus; NR, nonreactive; R, reactive; N, number; RHR, relative hazard ratio; OS, overall survival; HR, hazard ratio; CI, confidence interval.

We developed an algorithm for donor selection using classification and regression tree analysis of OS, incorporating NK cell alloreactivity predicted by CF-iKIR score and other donor characteristics. Classification and regression tree analysis identified four groups of donors by OS outcome (best, better, poor, and worst), as shown in **Figure 3**. Only 7% of transplants used donors who were older than 58 years and this group had the worst OS, regardless of other parameters. The best OS was found in CMV-nonreactive recipients with donors aged <58 years, which accounted for 12% of all transplants. In approximately 80% of transplants in which CMV-reactive recipients received a graft from donors aged <58 years, the CF-iKIR score significantly affected survival outcomes and was chosen as the best-split point to separate them into two large subgroups. The 3-year OS estimates were 73.9%, 54.1%, 44.5%, and 18.5% in the best, better, poor, and worst groups, respectively. Using the best donor group as a reference, the adjusted HRs for the better, poor, and worse groups were 1.84 (95% CI, 1.12-3.69; *P* = .044), 2.70 (95% CI, 1.41-5.19; *P* = .003), and 5.44 (95% CI, 2.54-11.64; *P* <.001), respectively. Internal validation using a dataset created from the bootstrapping method showed satisfactory predictive performance of this algorithm for OS, with a concordance index of 0.72.

## Discussion

In haplo-HSCT, unlike other forms of transplantation, a significant immune disparity exists in both HLA and KIR systems between donor and recipient, which may elicit the corresponding innate and adaptive alloreactivities. Recent studies have provided compelling evidence that the dynamics of NK cell recovery are remarkably affected by PTCy prophylaxis (24) and the amount of T cells in the graft (31). Models used in studies of matched unrelated donor HSCT were adapted to study KIR alloreactivity in haplo-HSCT, but findings have been discordant between studies (24, 26, 43). The use of haplo-HSCT is increasing, but HLA characteristics of an optimal donor are evolving (14), and the role of KIR-mediated alloreactivity remains unclear. In the present study, we thoroughly investigated the clinical effects of immunogenetic KIR models, along with other clinical factors, on outcomes of patients who received haplo-HSCT for hematologic malignancies. This single-institutional study showed that CF-iKIR score and other clinical variables were associated with outcomes of haplo-HSCT. These findings may aid in donor selection in haploidentical transplants and offer further critical insights into the role of KIR in patients receiving PTCy.

Contradictory results have been previously reported among studies of NK cell alloreactivity in TCR haplo-HSCT. It was initially believed that T cells contained in the graft may inhibit NK cell function and eliminate their GVL effect (44). Solomon et al. reported that mismatched KIR ligand and the presence of an active KIR haplotype (B/X with 2DS2) were associated with reduced relapse and superior disease-free survival (25). However, a recent study from the European Society for Blood and Marrow Transplantation (EBMT) that included 444 patients with acute leukemia showed that KIR ligand mismatch may be associated with a higher risk of relapse and was associated with significantly worse survival (26). The presence of B/x haplotype in the donor was reportedly associated with severe aGVHD (45). These conflicting results could reflect heterogeneity in the transplant protocol, graft source, KIR assumption or genotyping, and KIR alloreactivity models used.

Russo et al. extensively investigated the dynamics of NK cell recovery and concluded that most mature donor NK cells are eliminated by PTCy and therefore NK cell alloreactivity does not affect outcomes (24). A later study provided convincing evidence that in HSCT with incompatible KIR/HLA, alloreactive NK cells are particularly diminished by PTCy therapy (46). Moreover, NK cells derived from human stem cells are educated by HLA class-I molecules from both donor hematopoietic cells and host stromal cells (47), the potency of NK cell alloreactivity generated when the HLA ligands are missing on the recipient cells is therefore uncertain. In agreement with previous studies of haplo-HSCT with PTCy (24), we found that NK cell alloreactivity as predicted by the missing ligand model (donor NK cell benefit) or activating KIR/ligand model (KIR2DS1/C1C2 epitope combination), both of which have been widely investigated in studies of matched unrelated donor HSCT, did not affect outcomes.

In contrast to models that consider only the presence or absence of KIR and KIR ligands, the CF-iKIR score is an additive model incorporating multiple inhibitory KIRs and their corresponding ligands into an assigned score. In the present study, a CF-iKIR score >2 was associated with significantly better survival. The beneficial effect of this score is not in agreement with the currently preferred concept that NK cells would execute the antitumor effect when inhibitory KIRs miss the specific ligands or activating KIRs bind to the HLA ligands. Our results, however, could be in line with a few recent studies. In a large epidemiologic study, the CF-iKIR score predicted the incidence of viral infections in several independent cohorts (23), and high CF-iKIR scores were correlated with enhanced CD8^+^ T cell survival as well as a response against viral infections (23). Although most attention has been given to KIRs expressed on NK cells and NK cell-mediated anti-tumor effect, additional evidence has shown that inhibitory KIRs expressed on the T cells themselves may directly increase their lifespan (48–50). Additionally, NK cells may downregulate T cell response through certain cytokines or direct killing of activated T cells, but these negative regulations could be hindered by the presence of inhibitory KIRs (48, 51, 52). Moreover, a recent registry study also reported an association between CF-iKIR and superior event-free survival in patients with MDS or secondary AML although no significant relapse protection reported in that study was seen in this study (21).

In the present study, we found that a high CF-iKIR score was associated with improved OS and PFS, this could not be merely attributed to either lower NRM (HR, 0.69; *P* = .079) or relapse protection (HR, 0.94; *P* = .79) yet the combined effects on both could explain this finding. A registry study showed that higher donor age is associated with inferior mortality in haplo-HSCT (14), and our exploratory classification and regression tree analysis, which integrated donor characteristics and CF-iKIR scores, showed that donor age <58 years combined with a CMV-nonreactive recipient was associated with the best OS, whereas donor age ≥58 years was associated with the worst OS. In the rest of the cases (80% of our cohort), a high CF-iKIR score was associated with superior survival in CMV-reactive recipients whose donor was <58 years old, indicating that an interaction between donor CF-iKIR and recipient ligands may be protective against CMV reactivation. A previous study showed that a high CF-iKIR score is mostly associated with a protective effect against human immunodeficiency virus, hepatitis C virus, and human T-lymphotropic virus infections by augmenting CD8^+^ T cells (23). Nevertheless, the correlation between the CF-iKIR and CMV serostatus remains unclear, especially in the context of haplo-HSCT with PTCy. In the present study, we did not observe a significant effect of CF-iKIR on the total viral reactivation rate (data not shown). The anti-infective effect of inhibitory KIR ligand interaction, if there was any, might not be the predominant contributor to the improved survival observed in the present study.

Conflicting data have been published on the impact of donor-recipient CMV serostatus on outcomes of TCR haplo-HSCT. Although one study showed a protective effect of having a CMV-reactive donor with a CMV-reactive recipient (25), others have not reported any clinical relevance of donor CMV serostatus (53, 54). In the present study, we found that in all CMV-reactive recipients, the CF-iKIR score in each donor/recipient pair, regardless of donor CMV serostatus, was the salient factor in survival outcomes. In a heatmap comparison between all models of NK alloreactivity to date, we showed a minimal correlation between CF-iKIR and other models except with iKIR score (**Figure 1**) and none of the prior models described correlated significantly with survival except CF-iKIR score>2, which retained its significance in the multivariate model (**Figure 2**). Moreover, when this score was included in a CART analysis CF-iKIR score became the third most important factor influencing survival post-transplant, and an important factor to be considered in donor selection for patients receiving haplo-HSCT after donor age and CMV serostatus between donor and recipient (**Figure 3**). When combined with other factors, 4 groups of patients with very different survival ranging from 18.5% to 73.9% have been identified. Based on our findings, donor selection for haplo-HSCT can be summarized as follows: younger donors <58 years, preferably male (as older female donors are associated with higher NRM), CMV seronegative donor for CMV seronegative recipient, and with a CF-iKIR >2.

As an observational finding from a single institution study, the beneficial impact of CF iKIR in haplo-HSCT needs to be replicated and validated by an independent series of external studies. Our study has several limitations related to its retrospective nature and a relatively small number of patients from a single institution. Additionally, the lack of detailed longitudinal information on viral load and preemptive therapy for CMV limited our ability to accurately assess the impact of inhibitory KIR on CMV reactivation. Distinct KIR3DL1 subtype variants, combined with the specific HLA-B ligand subtype, could lead to various levels of inhibition on NK cells and relapse protection in AML patients after HSCT (18). This potential KIR variation may have been overlooked in our analysis because we did not include this model, which requires further KIR allele typing and HLA epitope assignment. Moreover, a recent study of HLA mismatch suggested that the alloreactivity derived from mismatch at individual HLA loci may differentially affect outcomes in haplo-HSCT (14). Given the limited number of patients in our cohort, HLA factors were not included to avoid overfitting in the multivariable models. However, influences from HLA mismatch that may be clinically important could therefore have been neglected in the present study. We would not recommend using CF iKIR score in the haploidentical donor selection at the current stage and a further registry-based multicenter study including these factors, as well as other confounding factors such as underlying disease, stem cell source, and conditioning intensity, is needed to verify our findings. Despite these limitations, using a relatively homogenous group of patients (treated the same, with <1% missing data), we found that NK alloreactivity appreciated by the CF-iKIR model is associated with survival in haplo-HSCT with PTCy, resulting in that mirror findings in unrelated donor transplantation. In addition, in CART analysis we found that this is one of the most important factors in donor selection for haploidentical transplants.

In conclusion, the present study showed that NK alloreactivity appreciated by CF-iKIR has a significant impact on the survival of patients receiving a haploidentical transplant and should be considered, in addition to other donor/recipient factors, in selecting donors for haploidentical transplantation.

## Data availability statement

The raw data supporting the conclusions of this article will be made available by the authors, without undue reservation.

## Ethics statement

The studies involving human participants were reviewed and approved by Institutional Review Board of The University of Texas MD Anderson Cancer Center. The ethics committee waived the requirement of written informed consent for participation.

## Author contributions

JZ, PK, SC, and KC designed the study and contributed to data collection and interpretation and manuscript writing; JZ and PK wrote the initial draft of the manuscript; PK contributed to statistical analysis and interpretation of statistical data; SAS, UG, SuS, and QM contributed to data collection and data analysis; JS, FH, and HB contributed to KIR model analysis and interpretation; BM contributed to KIR typing; YC and GR contributed to data collection; SAS, UG, KR, ES, RC, and SC contributed to the treatment of patients and reviewed and edited the final version of the manuscript. All authors contributed to the article and approved the submitted version.

## Funding

The research was partially funded by the departmental internal fund from the Department of Laboratory Medicine, MD Anderson Cancer Center (to KC and JZ).

## Acknowledgments

The authors would like to thank Dr. Jill Hollenbach from the University of California San Francisco for the helpful discussion on KIR analysis. We thank Erica Goodoff, Senior Scientific Editor in the Research Medical Library at The University of Texas MD Anderson Cancer Center, for editing this article.

## Conflict of interest

Authors JS, FH, HB are/were employed by DKMS gemeinnützige GmbH, Tübingen, Germany.

The remaining authors declare that the research was conducted in the absence of any commercial or financial relationships that could be construed as a potential conflict of interest.

## Publisher’s note

All claims expressed in this article are solely those of the authors and do not necessarily represent those of their affiliated organizations, or those of the publisher, the editors and the reviewers. Any product that may be evaluated in this article, or claim that may be made by its manufacturer, is not guaranteed or endorsed by the publisher.
