# Supplementary material for: Donor selection for KIR alloreactivity is associated with superior survival in haploidentical transplant with PTCy
Source: Front Immunol. 2022 Oct 13;13:1033871. doi: 10.3389/fimmu.2022.1033871 (PMC9606393; doi:10.3389/fimmu.2022.1033871)
Supplement: Supplementary file 1 [file DataSheet_1.pdf]

Supplemental Table 1. Percentage of patients in each KIR model in our cohort of patients who underwent haploidentical hematopoietic stem cell transplant (n = 354)*

	Donor NK cell benefit		KIR2DS1/C1C2 epitope combination		Donor centromeric motif			Donor telomeric motif			KIR B-content score			Inhibitory KIR score		CF-iKIR score	CF-iKIR score
	No	Yes	Neg	Pos	A/A	A/B	B/B	A/A	A/B	B/B	Neutral	Better	Best	≤2.5	>2.5	≤2	>2
Donor NK cell benefit				P=0.182			P=0.081			P=0.272			P=0.243		P<0.001		P<0.001
No			68.9	75.8	69.8	77	59.5	69.4	73.6	87.5	73	72.2	59.5	53.3	93.7	57.6	100
Yes			31.1	24.2	30.2	23	40.5	30.6	26.4	12.5	27	27.8	40.5	46.7	6.3	42.4	0
KIR2DS1/C1C2 epitope combination		P=0.182					P=0.007			P<0.001			P<0.001		P=1.000		P=0.483
Negative	60.5	68.3			69.2	59.3	43.2	93.5	4.7	0	76.8	7.4	43.2	62.6	62.9	61.3	65.5
Positive	39.5	31.7			30.8	40.7	36.8	6.5	95.3	100	23.2	92.6	56.8	37.4	37.1	38.7	34.5
Donor centromeric motif		P=0.081		P=0.007						P=0.010			P<0.001		P<0.001		P=0.300
A/A	50.2	54.5	56.8	42.4				53.9	50.9	18.8	68.1	5.6	0	64.1	35.9	52.5	49.1
A/B	41.1	30.7	36	41.7				36.2	35.9	81.3	31.9	94.4	0	21.5	58.5	35.7	43.1
B/B	8.7	14.9	7.2	15.9				9.9	13.2	0	0	0	100	14.4	5.7	11.8	7.8
Donor telomeric motif		P=0.272		P<0.001			P=0.010						P<0.001		P=0.945		P=0.006
A/A	63.6	70.3	97.8	11.4	68.7	62.2	62.2				79.5	0	62.2	64.6	66.7	63.9	69
A/B	30.8	27.7	2.3	76.5	29.7	28.2	37.8				20.5	70.4	37.8	30.8	28.9	29.4	31
B/B	5.5	2	0	12.1	1.7	9.6	0				0	29.6	0	4.6	4.4	6.7	0
KIR B-content score		P=0.243		P<0.001			P<0.001			P<0.001					P=0.016		P=0.076
Neutral	75.9	70.3	91	46.2	98.4	62.2	0	90.1	50.9	0				72.8	76.1	70.6	81.9
Better	15.4	14.9	1.8	37.9	1.7	37.8	0	0	38.9	100				12.8	18.2	17.7	10.3
Best	8.7	14.9	7.2	15.9	0	0	100	9.9	13.1	0				14.4	5.7	11.8	7.8
Inhibitory KIR score		P<0.001		P=1.000			P<0.001			P=0.945			P=0.016				P<0.001
≤2.5	41.1	90.1	55	55.3	68.7	31.1	75.7	54.3	56.6	56.3	54	46.3	75.7			81.9	0
>2.5	58.9	9.9	45	44.7	31.3	68.9	24.3	45.7	43.4	43.8	46	53.7	24.3			18.1	100
CF-iKIR score		P<0.001		P=0.483			P=0.300			P=0.006			P=0.076		P<0.001		
≤2	54.2	100	65.8	69.7	68.7	63	75.7	65.5	66	100	63.9	77.8	75.7	100	27		
>2	45.9	0	34.2	30.3	31.3	37	24.3	34.5	34	0	36.1	22.2	24.3	0	73		

Abbreviations: KIR, killer cell immunoglobulin-like receptor; NK, natural killer cell; CF-iKIR score, count functional inhibitory KIR score.
*All percentages are values of column variables.

Supplemental Table 2. Univariate analysis of the effect of baseline clinical characteristics on outcomes of haploidentical HSCT in our cohort (n = 354)

**Baseline factor**	**PFS**			**OS**			**Relapse**			**NRM**			**cGVHD**			**Viral reactivation**			**aGVHD grade 3-4**			**aGVHD grade 2-4**		
	**HR**	**95% CI**	**P**	**HR**	**95% CI**	**P**	**HR**	**95% CI**	**P**	**HR**	**95% CI**	**P**	**HR**	**95% CI**	**P**	**HR**	**95% CI**	**P**	**HR**	**95% CI**	**P**	**HR**	**95% CI**	**P**
Male	0.96	0.73-1.29	0.827	0.99	0.73-1.35	0.957	0.87	0.57-1.35	0.541	1.08	0.74-1.59	0.684	0.86	0.47-1.57	0.62	0.95	0.75-1.21	0.681	1.58	0.68-3.62	0.285	0.8	0.56-1.14	0.212
Sex mismatch																								
Other combination	Ref			Ref			Ref			Ref			Ref			Ref			Ref			Ref		
Female donor to male recipient	0.87	0.61-1.23	0.427	0.9	0.62-1.31	0.581	0.84	0.48-1.45	0.523	0.99	0.64-1.54	0.986	1.21	0.61-2.41	0.591	1.11	0.85-1.46	0.427	2.26	1.01-5.04	0.047	1.3	0.84-2.00	0.236
Recipient age, continuous	1.01	1.00-1.02	0.017	1.02	1.01-1.03	0.001	0.99	0.97-1.00	0.073	1.03	1.01-1.04	<0.001	0.99	0.97-1.01	0.342	0.99	0.98-1.01	0.474	1.00	0.98-1.04	0.559	0.99	0.98-1.01	0.385
HCT-CI, continuous	1.13	1.05-1.20	<0.001	1.15	1.07-1.23	<0.001	1.02	0.93-1.13	0.653	1.14	1.05-1.24	0.003	0.93	0.80-1.09	0.414	0.98	0.93-1.04	0.583	0.99	0.77-1.29	0.978	1.09	1.00-1.19	0.042
Disease risk index																								
Low or intermediate	Ref			Ref			Ref			Ref			Ref			Ref			Ref			Ref		
High or very high	2.03	1.52-2.70	<0.001	2.02	1.49-2.76	<0.001	2.48	1.60-3.86	<0.001	1.29	0.89-1.89	0.172	0.94	0.51-1.72	0.834	1.00	0.79-1.27	0.986	0.80	0.37-1.73	0.563	1.30	0.9101.87	0.151
Donor age, continuous	1	0.99-1.02	0.308	1.01	0.99-1.02	0.052	0.98	0.96-0.99	0.033	1.02	1.01-1.04	0.002	1.01	0.99-1.04	0.4	1.00	0.9901.01	0.273	1.03	1.00-1.06	0.025	1.01	0.99-1.02	0.276
ABO matching																								
Match	Ref			Ref			Ref			Ref			Ref			Ref			Ref			Ref		
Mismatch	0.97	0.72-1.32	0.862	1.05	0.76-1.44	0.784	0.80	0.50-1.30	0.373	1.13	0.76-1.67	0.548	0.88	0.45-1.72	0.715	0.92	0.72-1.17	0.483	1.60	0.74-3.48	0.235	0.92	0.62-1.37	0.693
Recipient-donor CMV status																								
NR-NR	Ref			Ref			Ref			Ref			Ref			Ref			Ref			Ref		

NR-R	1.10	0.42-2.89	0.849	1.30	0.44-3.85	0.642	1.28	0..35-4.74	0.712	0.91	0.23-3.67	0.9	0.49	0.05-4.53	0.527	0.99	0.46-2.17	0				1.46	0.46-4.60	0.521
R-NR	1.94	0.99-3.79	0.051	2.35	1.07-5.15	0.033	1.68	0.66-4.28	0.275	1.79	0.71-4.53	0.22	0.73	0.20-2.70	0.642	1.81	1.12-2.94	0.016	4.10	0.57-29.74	0.162	2.16	0.94-4.96	0.069
R-R	1.92	1.00-3.66	0.049	2.44	1.13-5.25	0.023	1.24	0.49-3.08	0.651	2.23	0.91-5.46	0.08	1.38	0.42-4.50	0.594	2.08	1.31-3.30	0.001				1.95	0.86-4.40	0.106
Stem cell type																								
BM	Ref			Ref			Ref			Ref			Ref			Ref			Ref			Ref		
PB	1.31	0.90-1.91	0.16	1.10	0.72-1.69	0.648	1.22	0.71-2.10	0.47	1.18	0.71-1.98	0.523	1.19	0.53-2.69	0.675	0.93	0.67-1.29	0.665	1.32	0.50-3.51	0.579	0.92	0.54-1.55	0.746
Conditioning regimen intensity																								
MA	Ref			Ref			Ref			Ref			Ref			Ref			Ref			Ref		
RIC/NMA	1.22	0.92-1.62	0.162	1.18	0.88-1.60	0.27	1.38	0.90-2.12	0.138	1.06	0.73-1.53	0.767	1.32	0.73-2.42	0.36	1.15	0.91-1.45	0.254	1.32	0.61-2.84	0.483	1.41	0.99-2.02	0.058
Donor specific anti-HLA antibodies (MFI>1,000)	1.32	0.84-2.08	0.23	1.3	0.81-2.10	0.281	1.36	0.70-2.65	0.36	1.11	0.59-2.11	0.742	0.73	0.22-2.44	0.607	0.96	0.66-1.40	0.825	2.48	0.92-6.29	0.071	0.91	0.47-1.77	0.778

Abbreviations: HSCT, hematopoietic stem cell transplant; PFS, progression-free survival; OS, overall survival; NRM, non-relapse mortality; cGVHD, chronic graft-versus-host disease; aGVHD, acute graft-versus-host disease; HCT-CI, hematopoietic cell transplant-comorbidity index; CMV, cytomegalovirus; NR, nonreactive; R, reactive; BM, bone marrow; PB, peripheral blood; MA, myeloablative; RIC, reduced-intensity conditioning; NMA, nonmyeloablative; HLA, human leukocyte antigen.

Supplemental Table 3. Multivariable analysis of the effect of the interaction of donor age and donor sex on risk of grade 3-4 aGVHD and NRM in patients who underwent haploidentical hematopoietic stem cell transplant (n = 354)

**Outcome**	**HR**	**95% CI**	**P**
Grade 3-4 aGVHD, adjusted by DSA			
Donor age <40 years, male	Ref		
Donor age <40 years, female	1.18	0.38-3.70	0.775
Donor age >40 years, male	1.58	0.53-4.69	0.409
Donor age >40 years, female	4.09	1.48-11.34	0.007
NRM, adjusted by recipient age and HCT-CI			
Donor age <40 years, male	Ref		
Donor age <40 years, female	1.09	0.65-1.81	0.755
Donor age >40 years, male	1.27	0.78-2.06	0.334
Donor age >40 years, female	2	1.15-3.46	0.014

Abbreviations: aGVHD, acute graft-versus-host disease; NRM, non-relapse mortality; HR, hazard ratio; CI, confidence interval; DSA, donor-specific antibodies; HCT-CI, hematopoietic cell transplant-comorbidity index.
